# Update on ocular manifestations of the main monogenic and polygenic autoinflammatory diseases

**DOI:** 10.3389/fopht.2024.1337329

**Published:** 2024-02-22

**Authors:** Alex Fonollosa, Ester Carreño, Antonio Vitale, Ankur K. Jindal, Athimalaipet V. Ramanan, Laura Pelegrín, Borja Santos-Zorrozua, Verónica Gómez-Caverzaschi, Luca Cantarini, Claudia Fabiani, José Hernández-Rodríguez

**Affiliations:** ^1^ Department of Ophthalmology, Biocruces Bizkaia Health Research Institute, Cruces University Hospital, University of the Basque Country, Barakaldo, Spain; ^2^ Department of Retina, Instituto Oftalmológico Bilbao, Bilbao, Spain; ^3^ Department of Ophthalmology, Rey Juan Carlos University Hospital, Madrid, Spain; ^4^ Department of Ophthalmology, Fundación Jiménez Díaz University Hospital, Madrid, Spain; ^5^ Research Center of Systemic Autoinflammatory Diseases and Behçet’s Disease Clinic, Department of Medical Sciences, Surgery and Neurosciences, University of Siena, Center of the European Reference Network (ERN) for Rare Immunodeficiency, Autoinflammatory and Autoimmune Diseases (RITA), Siena, Italy; ^6^ Allergy Immunology Unit, Department of Pediatrics, Advanced Pediatrics Centre, Postgraduate Institute of Medical Education and Research (PGIMER), Chandigarh, India; ^7^ Department of Paediatric Rheumatology, Bristol Royal Hospital for Children and Translational Health Sciences, University of Bristol, Bristol, United Kingdom; ^8^ Department of Ophthalmology, Institut Clínic d’Oftalmologia (ICOF), Hospital Clinic de Barcelona, University of Barcelona, Institut de Investigacions Biomediques August Pi i Sunyer (IDIBAPS), Barcelona, Spain; ^9^ Department of Biostatistics, Biocruces Bizkaia Health Research Institute, Bilbao, Spain; ^10^ Autoinflammatory Diseases Clinical Unit, Department of Autoimmune Diseases, Hospital Clínic de Barcelona, University of Barcelona, Institut d’Investigacions Biomèdiques August Pi i Sunyer (IDIBAPS), Center of the European Reference Network (ERN) for Rare Immunodeficiency, Autoinflammatory and Autoimmune Diseases (RITA), Spanish Center of the Centros, Servicios y Unidades de Referencia (CSUR) and Catalan Center of the Xarxa d’Unitats d’Expertesa Clínica (XUEC) for Autoinflammatory Diseases, Barcelona, Spain; ^11^ Ophthalmology Unit, Department of Medicine, Surgery and Neurosciences, University of Siena, Center of the European Reference Network (ERN) for Rare Immunodeficiency, Autoinflammatory and Autoimmune Diseases (RITA), Siena, Italy

**Keywords:** ocular inflammation, autoinflammatory diseases, monogenic autoinflammatory diseases, polygenic autoinflammatory diseases, conjunctivitis, uveitis, retinal vasculitis

## Abstract

Autoinflammatory diseases include disorders with a genetic cause and also complex syndromes associated to polygenic or multifactorial factors. Eye involvement is present in many of them, with different extent and severity. The present review covers ophthalmological lesions in the most prevalent monogenic autoinflammatory diseases, including FMF (familial Mediterranean fever), TRAPS (TNF receptor-associated periodic syndrome), CAPS (cryopyrin-associated periodic syndromes), Blau syndrome, DADA2 (deficiency of adenosine deaminase 2), DITRA (deficiency of the interleukin-36 receptor antagonist), other monogenic disorders, including several ubiquitinopathies, interferonopathies, and the recently described ROSAH (retinal dystrophy, optic nerve edema, splenomegaly, anhidrosis, and headache) syndrome, and VEXAS (vacuoles, E1 enzyme, X-linked, autoinflammatory, somatic) syndrome. Among polygenic autoinflammatory diseases, ocular manifestations have been reviewed in Behçet’s disease, PFAPA (periodic fever, aphthous stomatitis, pharyngitis and cervical adenitis) syndrome, Still’s disease and autoinflammatory bone diseases, which encompass CRMO (chronic recurrent multifocal osteomyelitis) and SAPHO (synovitis, acne, pustulosis, hyperostosis and osteitis) syndrome.

## Introduction

The term “autoinflammatory diseases” was used for the first time in 1999 to describe a group of rare diseases presenting with repeated episodes of uncontrolled systemic inflammation (1). Since then, the discovery of new monogenic diseases and other complex, multifactorial or polygenic autoinflammatory disorders, some of them sharing pathogenic features with autoimmune diseases and primary immunodeficiencies, but without a known genetic cause, have been in continuous expansion (2–4).

The most frequent and better characterized monogenic autoinflammatory diseases include familial Mediterranean fever (FMF), tumor necrosis factor (TNF) receptor-associated periodic syndrome (TRAPS), cryopyrin-associated periodic syndromes (CAPS), hyper-Immunoglobulin D syndrome (HIDS) and pediatric granulomatous arthritis (Blau syndrome and early onset sarcoidosis). Other monogenic disorders recently identified include retinal dystrophy, optic nerve edema, splenomegaly, anhidrosis, and headache (ROSAH) syndrome and vacuoles, E1 enzyme, X-linked, autoinflammatory, somatic (VEXAS) syndrome. The most well-recognized polygenic conditions include Behçet’s disease, periodic fever with aphthous stomatitis, pharyngitis and cervical adenitis (PFAPA) syndrome, Still’s disease, chronic recurrent multifocal osteomyelitis (CRMO), and synovitis, acne, pustulosis, hyperostosis and osteomyelitis (SAPHO) syndrome.

Clinical presentations in autoinflammatory diseases are highly variable and nonspecific, since most symptoms may overlap different conditions. Common manifestations include recurrent or persistent fever, musculoskeletal symptoms, abdominal and thoracic serosal pain, headache, and mucosal, cutaneous and ocular inflammatory lesions (5). With regard to the ophthalmological involvement in autoinflammatory diseases, all the ocular structures may be affected, including the eye surface (eyelid and conjunctiva), sclera, cornea, uvea, retina, optic nerve, lacrimal gland and extraocular muscles (5–7). Corneal degenerative disorders occasionally reported include keratoconus and retinal dystrophic lesions, such as retinitis pigmentosa-like disease. Cataract, glaucoma or choroidal neovascularization can also occur as complications due to a persistent and/or uncontrolled ocular inflammation (5–7).

The current review details the ocular presentations of the main monogenic and polygenic autoinflammatory diseases in order of prevalence and previous knowledge of their ophthalmologic involvement, which include eye acute and chronic manifestations, long-term complications, and therapeutic approaches of their systemic and ocular manifestations. All the monogenic and polygenic autoinflammatory diseases covered in this review and their main associated ocular manifestations are summarized in the **Tables 1**, **2**, respectively.

**Table 1:** Main monogenic autoinflammatory diseases with ophthalmological involvement and their ocular manifestations.

Monogenic autoinflammatory disease	Main acute ocular manifestations	Other described acute ocular lesions	Long-term ocular complications
Familial Mediterranean fever (FMF)	Uveitis (anterior, posterior, panuveitis), retinal vasculitis, conjunctivitis	Optic neuritis, papillitis, orbital myositis, scleritis, episcleritis, keratitis, periorbital edema, frosted branch angiitis, acute posterior macular placoid pigment epitheliopathy	Cataract, posterior synechiae, band keratopathy, cystoid macular edema, retinal detachment, corneal perforation/ectasia, oculomotor nerve palsy, decreased number of Meibomian glands
TNF receptor-associated periodic syndrome (TRAPS)	Conjunctivitis, periorbital edema	Multifocal choroiditis, panuveitis, optic neuritis	Cataract, posterior synechiae, glaucoma
Cryopyrin-associated periodic syndromes (CAPS)	Conjunctivitis, uveitis (anterior, intermediate, posterior and panuveitis)	Keratitis, episcleritis, periorbital edema, papillitis/papilledema, multifocal choroiditis, retinal vasculitis, retinitis, keratoendotheliitis fugax hereditaria	Posterior synechiae, cataract, band keratopathy, corneal leukoma, optic nerve atrophy, glaucoma, choroidal thickness reduction, retinal detachment, corneal perforation, retinal dystrophy, choroidal mass, nystagmus, ectopia pupillae, aphakia, keratoconus
Hyperimmunoglobulin D syndrome (HIDS)	Conjunctivitis, uveitis	Episcleritis, diffuse retinal hemorrhages, nummular keratitis	Cataracts, corneal scar, retinal dystrophy, retinal degeneration
Blau syndrome	Granulomatous uveitis (anterior and panuveitis)	Multifocal choroiditis, papilitis/papilledema, keratitis, episcleritis, ocular myositis, conjunctivitis, corneal ulcers	Cataract, glaucoma, posterior synechiae, band keratopathy, cystoid macular edema, retinal detachment, choroidal thickness reduction, corneal perforation, chorioretinal hypopigmentation/scars, choroidal neovascularization, macular edema, optic nerve swelling or atrophy
Deficiency of the interleukin-36 receptor antagonist (DITRA)	Lid excoriation and conjunctivitis	Scales and pustules on the lids	
NLRC4-associated autoinflammatory disease (NLRC4-AD)	Conjunctivitis, episcleritis, keratitis, dry eye	Anterior uveitis	
Deficiency of adenosine deaminase 2 (DADA2)	No predominant lesions	Strabismus, optic neuritis retinal vasculitis, anterior uveitis	Cataract, reduction in choroidal thickness
Haploinsufficiency of A20 (HA20)	Uveitis (anterior, intermediate, posterior)	Retinal vasculitis, episcleritis, aphthous ulcer of the palpebral conjunctiva	
RELA-associated inflammatory disease (RAID)	Conjunctivitis	Neuromyelitis optica	
Aicardi-Goutières syndrome (AGS)	Congenital glaucoma		Congenital glaucoma
STING-associated vasculopathy with onset in infancy (SAVI)	No predominant lesions		Glaucoma
Singleton-Merten syndrome (SMS)	No predominant lesions		Juvenile open-angle glaucoma
Chronic atypical neutrophilic dermatosis with lipodystrophy and elevated temperature (CANDLE)/Proteasome-associated autoinflammatory (PRAAS) syndrome	No predominant lesions	Posterior uveitis, violaceous puffy eyelids	
X-linked reticulate pigmentary disorder (XLRPD)	No predominant lesions	Photophobia	Corneal dyskeratosis and opacification
Retinal vasculopathy with cerebral leukodystrophy (RVCL)	Retinal arterial occlusions	Paracentral acute middle maculopathy	Retinal atrophy
Retinal dystrophy, optic nerve edema, splenomegaly, anhidrosis, and headache (ROSAH) syndrome	Optic nerve elevation with peripapillary thickening (on OCT), and macular edema	Retinal vasculitis, bilateral granulomatous anterior and intermediate uveitis with papillitis	Retinal degeneration (cone-rod dystrophy with atrophy). band keratopathy, cystoid macular edema
Vacuoles, E1 enzyme, X-linked, autoinflammatory, somatic (VEXAS) syndrome	Episcleritis, uveitis, scleritis, orbital/periorbital edema/inflammation with or without chemosis and pain	Retinal vasculitis, (orbital) inflammation of extraocular muscles, bulbar conjunctival hyperemia, proptosis, ptosis, epiphora, blepharitis, posterior scleritis, retinal detachment	

**Table 2:** Main polygenic autoinflammatory diseases with ophthalmological involvement and their ocular manifestations.

Polygenic autoinflammatory diseases	Main acute ocular manifestation	Other acute ocular lesions	Long-term ocular complications
Behçet’s disease	Panuveitis (anterior with hypopyon, and posterior with retinal vasculitis and multifocal retinitis)	Episcleritis, scleritis, keratitis, conjunctival ulcers, optic neuritis, oculomotor palsies, orbital inflammation	Cataract, glaucoma, epiretinal membranes, cystoid macular edema, optic atrophy, macular atrophy, retinal neovascularization,
Periodic fever, aphthous stomatitis, pharyngitis and cervical adenitis (PFAPA) syndrome	Periorbital pain, conjunctivitis	Intermediate uveitis, macular edema	Vitreous hemorrhage, tractional retinal detachment
Still’s disease			
Systemic juvenile idiopathic arthritis (sJIA)	No predominant lesions	Panuveitis	
Adult-onset Still disease (AOSD)	Purtscher’s-like retinopathy	Panuveitis, scleritis, throcleitis, orbital inflammation	
Autoinflammatory bone diseases			
Chronic recurrent multifocal osteomyelitis (CRMO)	No predominant lesions	Retinal vasculitis, optic neuropathy, central retinal artery occlusion, orbital inflammation	
Synovitis, acne, pustulosis, hyperostosis and osteitis (SAPHO) syndrome	No predominant lesions	Anterior scleritis, anterior uveitis, retinal vasculitis, Vogt-Koyanagi-Harada disease	

## Ocular manifestations in monogenic autoinflammatory diseases

### Familial Mediterranean fever

FMF is the first identified and most frequent monogenic autoinflammatory disease. It is caused by mutations in *MEFV* (Mediterranean FeVer), a gene encoding pyrin. A defective pyrin induces a constitutive activation of the inflammasome leading to an uncontrolled production of IL-1β and IL-18. Although FMF is primary inherited with an autosomal recessive pattern, in a remarkable proportion of patients the disease is transmitted with an autosomal dominant-like pattern. The most relevant pathogenic mutations (e.g. M694V, M694I, M680I and V726A) are located in the exon 10 of the *MEFV* gene (8).

FMF presentation is characterized by recurrent inflammatory attacks, usually self-limited in 8-72 hours, with a variable periodicity. Fever and abdominal pain due to sterile peritonitis are constantly present. Chest pain due to pleural inflammation, arthralgia involving large joints and cutaneous erysipeloid rash mostly affecting the limbs are also common. Other less frequent manifestations include pericarditis, febrile protracted myalgia, lymphocytic meningitis, and scrotal pain. Acute phase reactants levels, such as serum amyloid protein (SAA), C-reactive protein (CPR), and erythrocyte sedimentation rate (ESR), are typically increased during attacks and return to normal during asymptomatic periods. Secondary amyloidosis with renal involvement is the most common long-term complication, and is commonly associated with a more severe disease and a deficient control of disease activity (8).

Colchicine is the treatment of choice since it controls inflammatory symptoms, prevents the occurrence of new attacks and also the development of amyloidosis. Interleukin (IL)-1 blockers, such as anakinra and canakinumab, are effective in situations of intolerance or resistance to colchicine. Canakinumab is approved by the US Food and Drug Administration (FDA) and European Medicines Agency (EMA) for colchicine-resistant FMF. Other agents useful in isolated cases and small series with colchicine-resistant FMF include tocilizumab and tofacitinib (8, 9).

#### Eye disease

**Figure 1:**
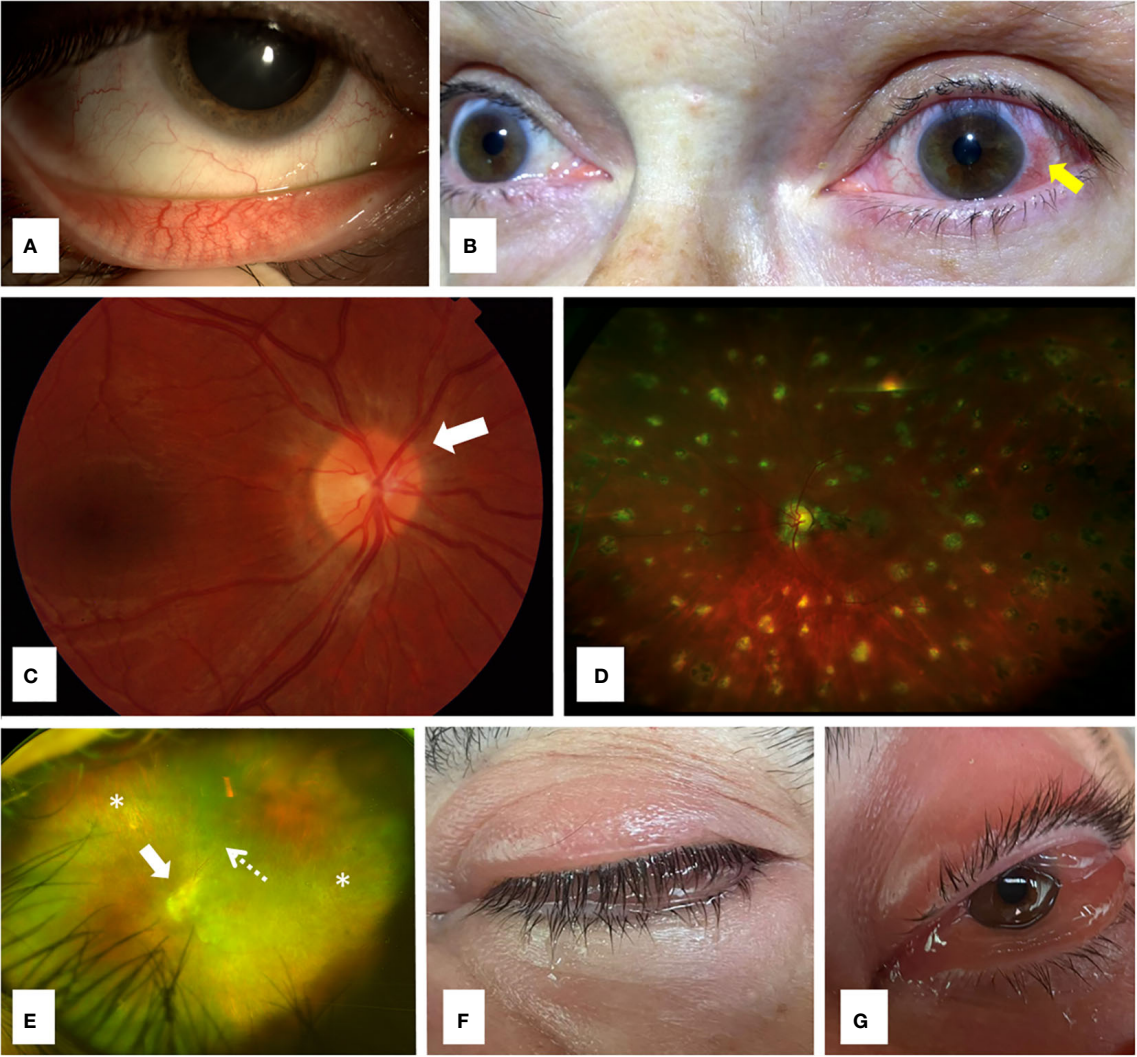
Ocular involvement in monogenic autoinflammatory diseases. **(A)** Conjunctivitis in a 32 years old female patient with familial Mediterranean fever; **(B)** Unilateral anterior uveitis with typical perilimbal hyperemia (arrow) in a 68 years old woman with Muckle-Wells syndrome, a cryopyrin-associated periodic syndrome (CAPS); **(C)** Fundoscopy revealing papilledema with optic nerve hyperemia (arrow) in a 36 years old woman with Muckle-Wells syndrome; **(D)** Ultra-wide field retinography showing diffuse lesions of multifocal choroiditis in a 17 years old male patient with Blau syndrome; **(E)** Ultra-wide field retinography displaying severe retinal atrophy (asterisks) and vessel attenuation (dashed arrow) with marked optic nerve swelling (arrow) in a 42 years old man with ROSAH syndrome, with long term loss of visual acuity and systemic symptoms; **(F)** Lid edema in a 72 years old male patient with VEXAS syndrome; **(G)** Prominent conjunctival chemosis due to orbital inflammation in the same patient **(F)** with VEXAS syndrome.

Ocular involvement in FMF is infrequent. In a Turkish series of 512 pediatric patients with FMF, eye disease was detected in 5 (1%) cases, and ocular lesions included bilateral anterior chronic uveitis, bilateral panuveitis, recurrent orbital myositis, recurrent optic neuritis, and acquired Brown’s syndrome (10). A literature review of 211 FMF patients with ophthalmological events showed that ocular manifestations occurred at a mean age of 19 years, after a mean age of 13 years at disease onset. Ocular lesions included retinal vasculitis in 31.3% and uveitis in 10.4% of patients (7). In order of frequency, uveitis was found anterior (6.6%), posterior (1.4%), intermediate (1%), and panuveitis (0.1%) (7). Frosted branch angiitis, a severe form of retinal vasculitis, have been occasionally reported in patients with FMF (11–13). Other less common ocular lesions described in FMF include posterior scleritis (14), acute posterior multifocal placoid pigment epitheliopathy (15), optic neuritis (16), papillitis, episcleritis and periorbital edema (7). In a multicentric Spanish cohort of adult and pediatric patients with FMF, conjunctivitis (**Figure 1A**), was present in two third of cases as the most prevalent ocular manifestation, followed by uveitis in 25% of cases, keratitis and episcleritis (16.7% each), and optic disk edema (8.3%). This observation was probably due to the high proportion of adult patients in this study, who probably exhibit milder forms of ocular disease (5), as it has been described for other systemic manifestations (17–21).

Structural optical coherence tomography (OCT) findings have been explored in FMF patients with contradictory results. While two series of pediatric patients with FMF experiencing recurrent attacks found a decreased choroidal thickness compared to age-matched controls (22, 23), other series of children with FMF showed no differences in choroidal thickness (24), and another study observed an increase in choroidal thickness in FMF attacks (25). OCT angiography findings have been also evaluated in FMF patients with conflicting results. Regarding retinal vascular plexus densities, a decreased vascular density in the deeper capillary plexus in 3x3 mm scans have been detected in FMF patients, with no differences in the remaining parameters analyzed (26). On the contrary, other authors reported no differences in the macular vessel density (either superficial or in deep capillary plexus) in FMF (27). In addition, a study found an increased vascular density in the temporal parafoveal quadrant of patients with FMF compared to controls (28).

Ophthalmologic complications in FMF are infrequent. A decreased number of Meibomian glands, without an increase in dry eye symptoms, has been described in pediatric patients with FMF (29). Corneal ectasia has been found more commonly in patients with FMF than in healthy individuals (30), and has been detected in 6.2% of FMF patients with ocular involvement (7). In this sense, corneal ectasia has been attributed to colchicine, as a probable cause of corneal wound healing impairment in these patients (31). While cataract and posterior synechiae have been reported in 8% of FMF cases presenting with ocular lesions (5), other ocular complications, such as band keratopathy, cystoid macular edema, retinal detachment, corneal perforation and oculomotor nerve palsy have been scarcely reported (7).

### TNF receptor-associated periodic syndrome

TRAPS is the most frequent autoinflammatory disease transmitted with an autosomal dominant pattern. It is caused by mutations in the *TNFRSF1A* (TNF Receptor Superfamily Member 1A) gene, which encodes TNF receptor 1. This protein malfunction induces a spontaneous production of IL-1β. The T50M variant and mutations affecting cysteine residues are common in children, since they are associated with an early onset, more severe presentation of the disease, and long-term complications, such as secondary amyloidosis, which has been reported in 25% of patients, mostly in those untreated or not well-controlled. Low penetrance mutations, such as R92Q and P46L, are generally associated with milder disease forms and later onset (8).

Disease onset typically occurs during childhood with recurrent and irregular febrile episodes with other inflammatory manifestations, mainly generalized myalgia, arthralgia, abdominal pain, skin rashes, and ocular and periorbital lesions (8). CRP, ESR and ferritin levels are usually increased during attacks and return to normal in asymptomatic periods (8).

Anti-IL-1 agents are the drugs of choice in TRAPS. While anakinra is effective in most cases, in continuous or on demand use, canakinumab is approved by the FDA and EMA as first line therapy in TRAPS. Among TNF blockers, etanercept has proved efficacy in controlling attacks. However, infliximab and adalimumab have been reported associated with severe (paradoxical) adverse effects in patients with TRAPS. IL-6 blockade with tocilizumab has shown benefit in some cases (8, 9).

#### Eye disease

Conjunctivitis and periorbital edema are the two most frequent ophthalmologic manifestations in TRAPS, since they have been reported to occur in 22% and 20%, respectively, in a series of 158 patients with TRAPS from the Eurofever registry. A literature review of 138 patients with TRAPS and ocular involvement similarly found conjunctivitis and periorbital pain/edema in 56.5% and 47.8% of them, respectively (7). In a Spanish series including 9 pediatric and adult patients with TRAPS and ocular complaints, conjunctivitis was the most frequent ophthalmologic manifestation, present in 88.9% of them, and periorbital edema was only observed in 11.1% of cases (5). In this regard, periorbital edema seem to occur more frequently in children than in adult patients, and in patients carrying structural mutations than in those with low-penetrant variants, such as R92Q (18, 32). However, in patients with R92Q-associated TRAPS and different ages at disease onset no differences in ocular manifestations have been observed (33).

Other less frequently reported ocular lesions in TRAPS include multifocal choroiditis, panuveítis and optic neuritis (7). With regard to ophthalmologic complications, cataract, posterior synechiae and glaucoma have been reported in less than 1% of patients with TRAPS and ocular involvement (5, 7).

### Cryopyrin-associated periodic syndromes

CAPS or cryopirinopathies are autosomal dominant autoinflammatory conditions caused by mutations in the *NLRP3* (Nucleotide-Binding Domain-Like Receptor Protein 3) gene, and encompass three disease forms presenting with different severity. The most severe form is the neonatal-onset multisystem inflammatory disease (NOMID) or chronic infantile, neurologic, cutaneous and articular (CINCA), the phenotype with intermediate severity is Muckle-Wells syndrome (MWS), and the mildest form is familial cold autoinflammatory syndrome (FCAS) (34). The *NLRP3* gene encodes NLRP3 or cryopyrin, and its malfunction leads to constitutive activation of NLRP3 inflammasome and uncontrolled IL-1β production. While pathogenic mutations generally cause severe disease presentations with neurologic complications and sensorineural hearing loss (8, 35), variants of uncertain significance and postzygotic mutations have been associated with milder disease forms (35). However, postzygotic variants in the *NLRP3* gene may also generate severe CAPS phenotypes in adult patients (35–39).

All CAPS forms have in common an early onset with inflammatory episodes consisting of fever, fatigue, urticarial rash, musculoskeletal symptoms and ocular inflammation. Acute phase reactants levels are usually increased during flares. FCAS attacks are classically triggered by cold exposure and tend to self-limit within less than 24 hours. MWS flares usually last 1-2 days, being sensorineural hearing loss and amyloidosis classical complications developed mostly in untreated or undertreated patients. CINCA/NOMID is characterized by a continuous systemic inflammation with persistent fever, urticarial lesions and severe osteoarticular, ocular and neurological involvement, usually leading to irreversible deformities and sequelae. Early initiation of a correct treatment in these patients is crucial to avoid mortality and disability (8).

IL-1 blockers are the first-line treatment for CAPS. In this sense, anakinra and canakinumab are approved by the FDA and the EMA for all forms of CAPS (8, 9).

#### Eye disease

Eye involvement has been reported in 71% of patients with CAPS in 136 cases included in the Eurofever Registry (34). MWS is the CAPS form more frequently associated with ocular manifestations, since they occur in 78% of subjects (40). In CAPS patients, systemic and ocular presentations usually occur sequentially at a mean age of 5.6 and 12 years, respectively (7). In a literature review of 680 patients (mostly pediatric) with CAPS, conjunctivitis was the most frequent ocular lesion, observed in 62.4% of cases, followed by uveitis (28.4%) (mainly anterior [**Figure 1B**]), papillitis or papilledema (**Figure 1C**) (27%), keratitis (10.6%), episcleritis (2.2%), multifocal choroiditis (1.9%), retinal vasculitis (1.3%), retinitis (0.4%) and periorbital edema (1%) (7). A recent series of 13 adult and pediatric patients showed conjunctivitis and uveitis as the main ocular lesions (present in 53.8% of them), followed by keratitis (38.5%), episcleritis (30.8%) and periorbital edema (7.7%) (5).

Keratoendotheliitis fugax hereditaria was described in 2018 as an isolated ocular condition associated to the missense mutation c.61G>C in the exon 1 of the *NLRP3* gene. It is characterized by unilateral attacks of keratitis, mainly affecting the endothelium, and occasional mild anterior uveitis resulting in stromal opacities (41). During flares, which seem to be triggered by cold wind or stress (42), corneal confocal microscopy detects hyperreflective structures suggesting inflammatory cells in the middle layers of corneal stroma (41).

Ocular complications in CAPS have been infrequently reported, and include optic nerve atrophy (7.4%), cataract (1.6%), band keratopathy (1.2%), glaucoma (0.6%), choroidal thickness reduction (0.6%), retinal detachment (0.3%) and corneal perforation (0.1%) (7). However, in a registry of adult and pediatric patients with CAPS and ocular manifestations, eye complications were observed in a higher proportion of cases, since 23% and 15.3% of them developed posterior synechiae and cataract, respectively. Band keratopathy and corneal leukoma were also complications occurring in 7% (each) of patients (5). Other sporadically reported eye lesions include retinal dystrophy, choroidal mass, nystagmus, ectopia pupillae, aphakia and keratoconus (7).

### Mevalonate Kinase Deficiency (MKD)

HIDS and mevalonic aciduria are two different phenotypes of mevalonate kinase (MVK) deficiency (MKD). Mevalonic aciduria corresponds to the most severe condition. Both diseases are caused by mutations in the *MVK* (Mevalonate Kinase) gene and transmitted in an autosomal recessive pattern. The *MVK* gene encodes MVK, an enzyme with a role on the non-steroidal isoprenoids synthesis and caspase activation pathway. Phenotype severity inversely correlates with the amount of residual enzymatic activity. V377I and I268T are the pathogenic mutations most commonly found. While most patients with HIDS carry heterozygous compounds, mevalonic aciduria is associated with homozygous I268T mutations (8).

Mevalonic aciduria is a neonatal disease presenting with recurrent attacks of fever, severe ocular, neurologic, and musculoskeletal manifestations, usually associated to growth retardation and dysmorphic features, and cutaneous lesions, hepatosplenomegaly, and lymphadenopathy. HIDS onset also occur during the first months of life and courses with monthly or bimonthly attacks of fever, oral aphthae, cervical or generalized lymphadenopathies, large joints arthralgia or non-erosive arthritis, abdominal pain and hepatosplenomegaly. Attacks may be triggered by infections, vaccines or trauma, and usually last from 3 to 7 days. Acute phase reactants, IgD and IgA levels are usually elevated during attacks. Increased mevalonic acid levels in urine during attacks may serve as a specific marker for MKD. Secondary amyloidosis is developed by about 3% of patients, mostly in those untreated or not treated properly (8).

Glucocorticoids at high doses usually control incompletely the attacks in patients with HIDS, and most of them require anti-IL-1 agents as first-line therapy. Anakinra can be successfully used continuously or on demand, and canakinumab is approved by the FDA and the EMA for patients with HIDS. Other biologics, such anti-TNF agents and tocilizumab, have been reported effective in some HIDS cases refractory to previous treatments (8, 9).

#### Eye disease

Ocular involvement in HIDS has been reported in 17% of patients (43). Conjunctivitis is the most frequent ocular lesion (in 10% of cases) followed by uveitis (2%) (5, 43). Other ophthalmologic lesions occasionally published include episcleritis (5), diffuse retinal hemorrhages (44), and nummular keratitis progressing to corneal scar (45). Cataract has been reported in 3% of patients with HIDS. Other described complications include retinal dystrophy (44, 46) and retinal degeneration (44).

### Blau syndrome

Pediatric granulomatous arthritis encompasses Blau syndrome and early-onset sarcoidosis. Both diseases are caused by autosomal dominant mutations in the *NOD2* (Nucleotide-Binding Oligomerization Domain-Containing Protein 2) gene, also known as CARD15 (Caspase Recruitment Domain-Containing Protein 15), which encodes NOD2. Blau syndrome is an autosomal dominant disease, and early-onset sarcoidosis is caused by the novo mutations, with no affected parents (8).

Both disorders have a disease-onset during the first ten years of life and are characterized by the sequential (although not constant) presentation of the triad of maculopapular rash, non-erosive arthritis and uveitis. Arthritis mainly affects wrists, hands, elbows and ankles. Fever, interstitial lung disease, large and small vessel vasculitis, cranial neuropathies and granulomatous involvement of spleen, liver, kidneys, and salivary glands are other less frequent manifestations. Although laboratory markers are usually normal, increased ESR and angiotensin-converting enzyme, and gammaglobulin levels may be observed (8).

Apart from glucocorticoids, TNF blockers are the most effective drugs in controlling disease activity in Blau syndrome. Anti-IL-1 and anti-IL-6 agents, and thalidomide, cyclosporine, methotrexate, and other conventional immunosuppressive drugs have been reported with some benefit (8).

#### Eye disease

Uveitis is the most common ocular manifestation in Blau syndrome, since it occurs in 95.4% of patients (7). Indeed, uveitis is part of the Blau syndrome classic triad (47, 48), which is completely present in more than half of patients (49). Blau syndrome accounts for 1.7% of all uveitis in Chinese patients (50). A literature review of 238 patients with Blau syndrome and ocular lesions found the age of ocular symptoms onset at a mean of 9 years, after a mean of 5 years from disease onset (7). Uveitis, mainly described as granulomatous, occurred in 95.4% of patients, being 48% anterior, 43.6% panuveítis, 11.9% posterior and 2.6% intermediate (7). Other authors have reported granulomatous bilateral panuveitis with multifocal choroiditis (**Figure 1D**) as the most common presentation in Blau syndrome (51, 52). Less frequent eye manifestations in this large literature review included multifocal choroiditis (15.1%), papillitis/papilledema (10.9%), keratitis (6.3%), episcleritis (1.3%), and ocular myositis (0.4%) (7). Conjunctivitis (5) and corneal ulcers due to trigeminal neuropathy (53) have been also reported in Blau syndrome. The development of optic nerve swelling or atrophy is caused by the acute and/or persistent inflammation (51, 52).

Patients with Blau syndrome present with the highest rate of ophthalmologic complications among all monogenic autoinflammatory diseases (5). These complications are usually associated with the severity of uveitis (5, 7). In a literature review, late complications in Blau syndrome included cataract (23.9%), glaucoma (9.7%), cystoid macular edema (8%), retinal detachment (1.7%) and optic atrophy (3.8%). Other less frequent ocular complications were choroidal thickness reduction, corneal perforation, and chorioretinal hypopigmentation (7). Other authors have found cataract, posterior synechiae, glaucoma and band keratopathy in a higher proportion of patients with Blau syndrome (5). Severe lesions, such as choroidal neovascularization, chorioretinal scars and macular edema, have been also described in patients with Blau syndrome (5).

### Deficiency of the interleukin-36 receptor antagonist

DITRA is an autosomal recessive autoinflammatory disease considered a form of generalized pustular psoriasis. It is caused by mutations in the *IL36RN* (Interleukin 36 Receptor Antagonist) gene, which encodes IL-36 receptor antagonist (IL36Ra). This protein malfunction induces the NF-κB activation and the overproduction of proinflammatory cytokines, such as IL-36 and IL-8 (8).

DITRA may have a disease onset at pediatric and adult ages. The clinical picture consists of episodes of high-grade fever, skin lesions of generalized pustulosis and asthenia. Attacks can be triggered by infections, pregnancy and menstruation. Characteristic laboratory features include increased levels of acute phase reactants and leukocytosis (8).

Treatment of DITRA includes topical and systemic therapies regularly used for psoriasis. Anti-IL-1 agents, such as anakinra, TNFα blockers, ustekinumab (anti-IL-12/23) and secukinumab (anti-IL-17) have been reported effective in isolated cases (8). Spesolimab, an interleukin IL-36 receptor antagonist was approved by the FDA in 2022 and conditionally approved by EMA in 2023 for the treatment of generalized pustular psoriasis flares in adults based on the positive results of a randomized, double-blind, multicenter, phase II clinical trial (NCT03782792) (54).

#### Eye disease

Ocular involvement in psoriasis vulgaris usually affects the lid, conjunctiva, cornea and anterior uveal tract. Although no patients with genetically confirmed DITRA and ophthalmologic lesions have been published to date, a single patient diagnosed with generalized pustular psoriasis and Sjögren’s syndrome (without a genetic study for DITRA) has been reported presenting with eye lesions consisting in lid excoriation and conjunctivitis, and also with a periocular form of pustular psoriasis combining scales and pustules on the lids (55).

### NLRC4-associated autoinflammatory disease

NLRC4-AD is an autosomal dominant autoinflammatory disease caused by mutations in the *NLRC4* (NLR family CARD domain-containing protein 4) gene, and encompasses two different phenotypes: NLRC4-associated macrophage activation syndrome (NLRC4-MAS) and familial cold autoinflammatory syndrome 4 (FCAS4). The encoded protein, NLRC4, leads to NLRC4 inflammasome activation, which results in an increased secretion of IL-1 and IL-18 (8).

NLRC4-MAS is the most severe form, usually initiated during the first year of life and characterized by chronic inflammatory bowel disease, MAS, or symptoms mimicking CINCA/NOMID. Enterocolitis manifestations tend to subside over time. FCAS4 is the milder form, usually initiated during childhood with attacks of urticaria, arthralgia, and ocular inflammation after cold exposure. Fever is present in half of cases. With regard to laboratory features, CRP levels are elevated during attacks in severe cases, and ESR values tend to decrease as the disease worsens. IL-18 levels tend to be particularly high in patients with NLRC4-MAS, and may persist elevated even in the absence of clinical activity (8).

Glucocorticoids and anakinra may be useful in some mild cases. IL-18 blockade and anti-interferon-gamma (IFNγ) inhibition have been reported effective in severe cases (8).

#### Eye disease

In a series of 26 patients with NLRC4-AD, conjunctivitis, episcleritis, keratitis, dry eye have been reported in 42% of patients, and were only present in the FCAS4 subgroup (61.1%), while no cases were found in patients with NLRC4-MAS (56). Anterior uveitis has been also reported in a patient with NLRC4-AD (5).

### Deficiency of adenosine deaminase 2

DADA2 is an autoinflammatory disease inherited in an autosomal recessive pattern. It is caused by mutations in the *ADA2* (Adenosine Deaminase 2) gene, which encodes ADA2, a growth factor in myeloid cells that stimulates differentiation into anti-inflammatory macrophages, and plays a role in the development and maintenance of endothelial cells. ADA2 dysfunction promotes vascular damage. Although disease typically occurs in early childhood, adult-onset cases have been also reported (8).

DADA2 presentation may be indistinguishable from polyarteritis nodosa (PAN), since it is characterized by persistent or recurrent fever, skin livedoid or nodular lesions, peripheral neuropathy, and vascular lesions causing distal ischemia or hemorrhage of the brain and other territories. Oral aphthae, musculoskeletal complaints and hepatosplenomegaly are also frequent. Laboratory features include increased acute phase reactants levels during attacks and changes suggesting certain degree of immunodeficiency, such as low immunoglobulin levels and peripheral blood cytopenias (8).

Glucocorticoids, usually at high-to-moderate doses, control disease activity in DADA2. Among all conventional and biologic immunosuppressive drugs, only anti-TNF agents, and in particular etanercept, have demonstrated to be effective in controlling inflammatory manifestations and progression of vascular disease. Allogeneic hematopoietic stem cell transplantation is the only therapy currently able to cure DADA2 (8).

#### Eye disease

In DADA2, a reduction in choroidal thickness, strabismus, retinal vasculitis, optic neuritis (7) and anterior uveitis (5) have been observed in isolated cases. A patient with DADA2 and anterior uveitis, also developed cataract (5).

### Haploinsufficiency of A20

HA20 is an autosomal dominant autoinflammatory disease caused by mutations in the *TNFAIP3* (TNF Alpha Induced Protein 3) gene encoding protein A20. Loss-of-function mutations in *TNFAIP3* result in insufficient suppression and subsequent increased NF-κB signaling, and NLRP3 constitutive activation, both leading to an overproduction of proinflammatory cytokines, such as IL-1, IL-6, IL-18 and TNF-α (8, 57).

HA20 is also known as monogenic Behçet-like disease, since it is clinically characterized the triad of orogenital ulcers, ocular inflammation and non-deforming polyarthritis. Other manifestations include pharyngitis, pericarditis, abdominal pain, central nervous system vasculitis and ocular inflammation. Several organ-specific and systemic autoimmune diseases, such as type 1 diabetes mellitus, Hashimoto thyroiditis, neutrophilic dermatosis, pseudofolliculitis, erythema nodosum, central nervous system vasculitis, immunoglobulin A vasculitis, Kawasaki disease, idiopathic thrombocytopenic purpura nephrotic syndrome, or interstitial lung disease, have been associated with HA20. Laboratory and immunological changes include high acute phase reactants levels during flares and low-titer autoantibodies, mainly in patients with concomitant autoimmune diseases (8, 57).

Glucocorticoids, colchicine, mesalazine, methotrexate, azathioprine, cyclosporine and thalidomide have been useful in some patients. Anti-TNF, anti-IL-1 and anti-IL-6 agents have been reported effective in some patients with HA20 refractory to previous treatments (8, 57).

#### Eye disease

Bilateral or unilateral uveitis (anterior, intermediate, and posterior, including retinal vasculitis), episcleritis, and an aphthous ulcer of the palpebral conjunctiva have been reported in patients with HA20 (57–62).

### RELA-associated inflammatory disease

RAID, also known as RELA haploinsufficiency, is an autosomal dominant autoinflammatory disease caused by mutations in the *RELA* (V-Rel Avian Reticuloendotheliosis Viral Oncogene Homolog A) gene, which encodes RelA or p65 protein. This protein is a subunit of the transcription factor NF-κB. The NF-kB pathway plays a crucial role in immunity and inflammation, and RELA is an essential regulator of mucosal homeostasis. Therefore, RELA haploinsufficiency leads to decrease NF-κB signaling and to stimulate TNF-driven mucosal apoptosis with impaired epithelial recovery. Patients with RAID exhibit mucosal abnormalities without immunodeficiency (8, 63).

The clinical picture of RAID consists in orogenital ulcers, rash, joint inflammation, and fever. Some patients present with gastrointestinal symptoms, such as abdominal pain, vomiting and diarrhea. Disease phenotypes can resemble Behçet’s disease and/or inflammatory bowel disease. Acute phase reactants tend to be high during flares. Glucocorticoids, colchicine and TNF inhibitors have shown efficacy in patients with RAID (8, 63).

#### Eye disease

Conjunctivitis has been described in 13% of cases in a series of 15 patients with RAID (63). Neuromyelitis optica has been also reported in a member of a family with RAID (64).

### Aicardi-Goutières syndrome

AGS comprises a group of seven monogenic autoinflammatory diseases included in the group of interferonopathies. The majority of them have an autosomal recessive transmission. Responsible genes (*TREX1* [Three Prime Repair Exonuclease 1], *RNASEH2A* [Ribonuclease H1 2A], *RNASEH2B, RNASEH2C, SAMHD1* [SAM and HD Domain Containing Deoxynucleoside Triphosphate Triphosphohydrolase 1], *ADAR1* [Adenosine Deaminase RNA Specific], *IFIH1* [Interferon Induced with Helicase C Domain 1] and *DDX58* [DExD/H-box Helicase 58]) encode proteins involved in intracellular degradation or sensing of nucleic acids. Their malfunction induces high levels of IFNα, both in blood and cerebrospinal fluid, which play a pivotal role in the systemic and cerebral tissue damage typical of AGS (8).

Shared features in all forms of AGS include neonatal-onset encephalopathy (with basal ganglia calcifications, spasticity, dystonia, progressive cerebral atrophy and microcephaly), fever and hepatosplenomegaly. Lymphocytosis and elevated IFNα levels in cerebrospinal fluid are characteristic laboratory changes. Patients with AGS may present with autoimmunity features resembling systemic lupus erythematosus, such as arthritis, lymphopenia, thrombocytopenia and antinuclear antibodies (ANA) positivity (8).

Glucocorticoids, conventional immunosuppressive drugs and several anti-cytokine (e.g. anti-TNF, anti-IL-1 and anti-IL-6) agents are not effective in AGS. As in other interferonopathies, Janus kinase (JAK) inhibitors are the treatment of choice of AGS (8).

#### Eye disease

Ocular involvement in AGS is characterized by the presence of congenital glaucoma due to the dysgenesis of the anterior segment of the eye (65–68).

### STING-associated vasculopathy with onset in infancy

SAVI is an autosomal dominant interferonopathy caused by mutations in the *STING* (Stimulator of Interferon Genes) gene, also known as *TMEM173* (Transmembrane Protein 173) gene, which encodes STING. This protein is an indirect sensor of cytosolic DNA activating IRF3 and inducing transcription of IFN-1 related genes. STING malfunction results in overactivation of IRF3 and uncontrolled transcription of IFNβ (8).

SAVI is manifested during the neonatal period with recurrent attacks of fever, cutaneous rash, small-vessel vasculitis and interstitial lung disease. Acute phase reactants are elevated during flares and low-titer autoantibodies, such as ANA, antineutrophil cytoplasmic antibodies (ANCA), and antiphospholipid antibodies, are frequently detected. As in other interferonopathies, baricitinib and other JAK inhibitors are effective in SAVI (8).

#### Eye disease

Glaucoma has been reported in patients with SAVI (69).

### Singleton-Merten syndrome

SMS is an interferonopathy inherited with an autosomal dominant pattern and caused by mutations in *IFIH1* or *DDX58* genes, which encode melanoma differentiation associated protein 5 (MDA5) and retinoic-acid-inducible gene I (RIG-I), respectively. Both proteins are involved in type I IFN induction pathways (8).

Neonatal clinical manifestations include dental dysplasia, tendon rupture, arthropathy, osteoporosis, aortic calcification, neurologic abnormalities, glaucoma and localized or generalized psoriasis. JAK inhibitors are the drugs of choice in SMS (8).

#### Eye disease

Regarding ocular lesions, only juvenile open-angle glaucoma has been reported in SMS patients (65, 70).

### Chronic atypical neutrophilic dermatitis with lipodystrophy and elevated temperature syndrome

CANDLE syndrome, also known as proteasome associated autoinflammatory syndrome (PRAAS), is an autosomal recessive interferonopathy caused by mutations in the *PSMB8* (Proteasome 20S Subunit Beta 8) gene, which encodes the β5i subunit of the immunoproteasome. Other identified proteasome genes causing CANDLE/PRAAS are *PSMB9, PSMA3* (Proteasome 20S Subunit Alpha 3), *PSMB4* and *POMP* (Proteasome Maturation Protein). Dysfunctional genes cause defective proteasome/immunoproteasome assembly and accumulation of ubiquitinated proteins, which in turn induce intracellular stress by increasing IFN-1 production through JAK signaling pathway (8).

The clinical picture of CANDLE/PRAAS is characterized by a neonatal onset with recurrent or persistent fever, cutaneous lesions, and facial and generalized lipodystrophy. Other common manifestations include muscle atrophy, arthralgia, lymphadenopathy, hepatosplenomegaly, and inflammatory involvement of other territories, such as meninges, epididymis, parotids and eyes. Elevated acute phase reactants levels are constant, muscle and hepatic enzymes are frequently increased, and ANA and ANCA may be present in some patients (without clinical relevance) (8).

As in other interferonopathies, glucocorticoids, conventional and biologic immunosuppressive drugs, such as anti-IL-1, anti-TNF or anti-IL-6 have been used with null or incomplete response. Baricitinib has shown efficacy in patients with CANDLE/PRAAS (8).

#### Eye disease

Posterior uveitis and violaceous puffy eyelids have been reported in 2 and 1 patients with CANDLE/PRAAS, respectively (71).

### X-linked reticulate pigmentary disorder

XLRPD is an X-linked genetic disease caused by an intronic mutation in *POLA1* (DNA Polymerase Alpha 1, Catalytic Subunit) gene, which encodes the catalytic subunit of DNA polymerase-α (Pol-α). Reduced POLA1 expression triggers spontaneous type I interferon expression, and is also associated with a decreased number and selective cytotoxicity defect of NK cells.

XLRPD is characterized by recurrent infections and different clinical and histological features depending on the sex. Males manifest a prominent reticulate hyperpigmentation with mottled hypopigmentation and typical facial features, such as upswept frontal hairline and flared eyebrows, with additional systemic manifestations, such as hypohidrosis gastrointestinal inflammation, recurrent respiratory infections, failure to thrive, and photophobia due to corneal opacification. Heterozygous females have milder disease consisting of brown patchy pigmentary skin lesions along the lines of Blaschko (secondary to the clonal proliferation of keratinocytes developed from postzygotic mutations), without systemic manifestations (72, 73).

JAK inhibition with tofacitinib has been reported useful in a patient with XLRPD (74).

#### Eye disease

Males affected by XLRPD may occasionally present with photophobia due to corneal dyskeratosis and opacification (73, 75).

### Retinal vasculopathy with cerebral leukodystrophy

RVCL is an autosomal dominant autoinflammatory disease caused by mutations in the *TREX1* gene, which encodes a 30-50 DNA exonuclease involved in clearing cytosolic nucleic acids. RVCL covers three neurovascular syndromes previously described as cerebroretinal vasculopathy (CRV), hereditary vascular retinopathy (HVR), and hereditary endotheliopathy, retinopathy, nephropathy and stroke (HERNS) (76). Other diseases caused by *TREX1* mutations are known interferonopathies, such as monogenic systemic lupus erythematosus, Aicardi-Goutières syndrome and familial chilblain lupus.

RVCL affects adults between 35 and 50 years of age with predilection by small vessels in different tissues including the retina, brain, liver, kidney and. Retinal involvement is constant, and may precede the central nervous system involvement. Neurologic manifestations include hemiparesis, aphasia, hemianopsia, facial palsy, and less frequently seizures and migraines. Kidneys are affected by mild-to-moderate renal function impairment and mild proteinuria, and rarely progresses to end-stage kidney disease. Hepatic abnormalities are characterized by mildly elevated alkaline phosphatase and gamma-glutamyl transferase levels. Less common features include Raynaud phenomenon, hypertension, psychiatric disorders, and anemia (76, 77).

Symptomatic treatment is the only option currently available. No use of JAK inhibitors has been reported. Ophthalmologic therapy is similar to that used in diabetic retinopathy. Patients may require retinal laser photocoagulation (mostly for peripheral retina), and intravitreal anti-vascular endothelial growth factor therapy. Treatment for secondary glaucoma may be also required (77).

#### Eye disease

Decreased visual acuity and/or visual field defects are the most frequent manifestations in RVCL due to the involvement of the small retinal vessels typically producing retinal vasculopathy associated to areas of artery occlusions, arterial attenuation, and cotton wool spots (78–80). OCT in patients with RVCL usually discloses retinal thinning in the peripapillary and macular area (77). Areas of paracentral acute middle maculopathy can be also detected by OCT (81).

### Retinal dystrophy, optic nerve edema, splenomegaly, anhidrosis, and headache syndrome

ROSAH syndrome was described in 2019 as an autosomal dominant autoinflammatory disease, caused by mutations in the *ALPK1* (Alpha Kinase 1) gene, which encodes ALPK1 (82). This protein acts as a sensor for bacterial sugars, and *in vitro* studies have showed that mutated ALPK1 confers an activation of the NF-κB signaling pathway, STAT1 phosphorylation and interferon gene expression signature (82, 83). This syndrome was initially reported in 2012 in a family with splenomegaly, cytopenias, and vision loss (84).

Ocular manifestations in ROSAH syndrome are accompanied by systemic features including recurrent low-grade fever, malaise, arthralgia, deforming arthritis, abdominal pain, headache due to meningeal inflammation, and premature cerebral mineralization of the basal ganglia, substantia nigra and red nuclei. Other clinical manifestations include multiple dental caries with hypoplasia of dental enamel and short dental roots, dry mouth, inability to sweat or lactate and splenomegaly (83, 85). Laboratory changes are characterized by a highly variability in CRP levels in untreated patients, and transitory increases in CRP levels without correlation with disease activity. High CRP values are typically associated with transient cytopenias, mainly neutropenia (reversible after splenectomy) and lymphopenia, without an increased susceptibility for bacterial infections (83).

Oral glucocorticoids have shown efficacy in the control of intraocular inflammation in some patients. Among anti-cytokine agents, tocilizumab has been reported with the highest efficacy in controlling ocular and systemic manifestations, even in cases refractory to conventional immunosuppressive drugs, and anti-IL-1/TNF agents. The potential effect of JAK inhibitors has not been proven yet (83, 85).

#### Eye disease

A recent series of 11 patients with ROSAH syndrome, with 7.3 to 60.2 years at the time of the initial evaluation, found a best-corrected visual acuity at baseline ranging from 20/16 to no light perception. Intraocular inflammation sequelae were observed in 9 patients, including keratic precipitates, band keratopathy, presence of cells in the anterior chamber, and cystoid macular edema, as well as retinal vasculitis on fluorescein angiography. Ten (90.1%) patients had and elevation of the optic disc with peripapillary thickening on OCT, and 7 (63.6%) patients showed retinal degeneration (**Figure 1E**) consistent with a cone-rod dystrophy, with atrophy involving the posterior pole with a peripheral extent. One patient with a normal electroretinography and visual evoked potential results had decreased Arden ratio on electrooculography. This study identified three principal risk factors for visual deterioration in patients with ROSAH syndrome: a) optic nerve involvement; b) intraocular inflammation (including cystoid macular edema); and c) retinal degeneration (85). Bilateral granulomatous anterior and intermediate uveitis with papillitis associated with retinal dystrophy features have been also described in a patient with ROSAH syndrome (86).

### Vacuoles, E1 enzyme, X-linked, autoinflammatory, somatic syndrome

VEXAS syndrome was described in 2020 as an autoinflammatory disease caused by postzygotic variants in the *UBA1* (Ubiquitin Like Modifier Activating Enzyme 1) gene, located on the short arm of the X chromosome (87). A mutated UBA1 protein produces a dysfunction of the E1 enzyme and disruption of the ubiquitination process, with an incomplete protein ubiquitination and degradation by the cytoplasmic proteasome complex. These aberrant products lead to cellular stress and activation of different pathophysiological mechanisms, such as the NF-κB transcription factor pathway with increase of TNFα, IL-6 and IFNγ in circulating monocytes and neutrophils. VEXAS syndrome occurs in adult males, although it has been also described in a few number of women, usually with X monosomy (87, 88).

Clinical manifestations include recurrent fever, arthralgia and arthritis, ear and nose chondritis, cutaneous involvement, mainly as neutrophilic dermatoses, pulmonary infiltrates, venous thrombotic events, and different types of vasculitis. Elevated acute phase reactants and macrocytic anemia are constantly observed. A concomitant occurrence of myelodysplasia is frequent, and bone marrow cytoplasmic vacuoles in myeloid and erythroid cell precursors are highly suggestive of this disease (88, 89).

Glucocorticoids are only effective at medium-high doses, and the use of many other immunosuppressive (conventional or biological) drugs has showed limited or null efficacy. Hypomethylating agents, such as azacitidine, have been associated with good responses, especially in patients presenting with myelodysplastic syndrome. Currently, allogeneic hematopoietic stem cell transplantation seems to be the only curative therapy (88, 89).

#### Eye disease

Ocular involvement in VEXAS syndrome has been described to occur in 28% to 55% of patients (87, 90, 91). In a French series of 116 patients with VEXAS, 40% of them had eye lesions, which in order of frequency included episcleritis (12.1%), uveitis (9.5%), scleritis (8.6%), periorbital edema (8.6%) and orbital mass (3.4%) (91). The same French group analyzed the differences between patients with relapsing polychondritis-associated to VEXAS syndrome and isolated relapsing polychondritis and found a higher ocular involvement in patients with relapsing polychondritis and VEXAS (57% vs. 11%), with a slightly higher frequency of uveitis (17% *vs*. 5%) and episcleritis (28% *vs*. 15%), and a similar proportion of scleritis (13% *vs*. 10%) and retinal vasculitis (4% *vs*. 3%) (92).

With regard to orbital inflammation, it has been reported from 8% to 16% of cases with VEXAS syndrome (87, 91, 93). Orbital and periorbital involvement with (or without) inflammation of extraocular muscles producing eye pain, palpebral edema/hyperemia (**Figure 1F**), bulbar conjunctival hyperemia with chemosis (**Figure 1G**), proptosis, ptosis, and epiphora (resembling an orbital cellulitis or panniculitis) have been described in several patients with VEXAS (93–96). Blepharitis, posterior scleritis (93) and retinal detachment (97) have been also reported in patients with VEXAS.

## Ocular manifestations of polygenic autoinflammatory diseases

### Behçet’s disease

Behçet’s disease is a chronic systemic disease classified as both polygenic autoinflammatory disease and variable vessel vasculitis, because arteries and veins of any size can be involved (98). Environmental factors, such as infectious agents, are thought to trigger the inflammatory manifestations by inducing an aberrant immune response in genetically susceptible individuals. It is more prevalent in the area from Eastern Asia to the Mediterranean sea (98).

Behçet’s disease is characterized by recurrent episodes of mucocutaneous, ocular, musculoskeletal, vascular, gastrointestinal, and central nervous system involvement. Mucocutaneous manifestations include non-scarring oral ulcers, genital ulcers, papulopustular, pseudo-folliculitis or acneiform lesions, superficial venous thrombophlebitis and erythema nodosum like lesions (99).

The European League Against Rheumatism (EULAR) recommendations for the management of Behçet’s disease include the use of glucocorticoids and colchicine for mucocutaneous and articular manifestations. Glucocorticoids and other immunosuppressive drugs, such as cyclophosphamide, can be used to treat severe manifestations, such as acute deep vein thrombosis and pulmonary aneurysms. In refractory cases, TNF-blockers are recommended. Eye involvement has to be treated according to the severity of the ocular lesions. Mild cases can be treated with glucocorticoids in combination with azathioprine, cyclosporine-A, IFNα or anti-TNF agents. In patients with an initial or recurrent sight-threatening uveitis, high-dose glucocorticoids, infliximab or IFNα are recommended. Intravitreal glucocorticoid injections can be additionally administered in patients with unilateral exacerbations (100, 101).

#### Eye disease

Uveitis is the most frequent ophthalmological manifestation in Behçet’s disease. Indeed, uveitis is one of the major classificatory criteria, together with oral and genital ulcers, skin lesions and pathergy test. It is usually bilateral and non-granulomatous in nature, and panuveitis is the most frequent type of intraocular inflammation. Presentation of uveitis is typically acute and explosive (102, 103).

**Figure 2:**
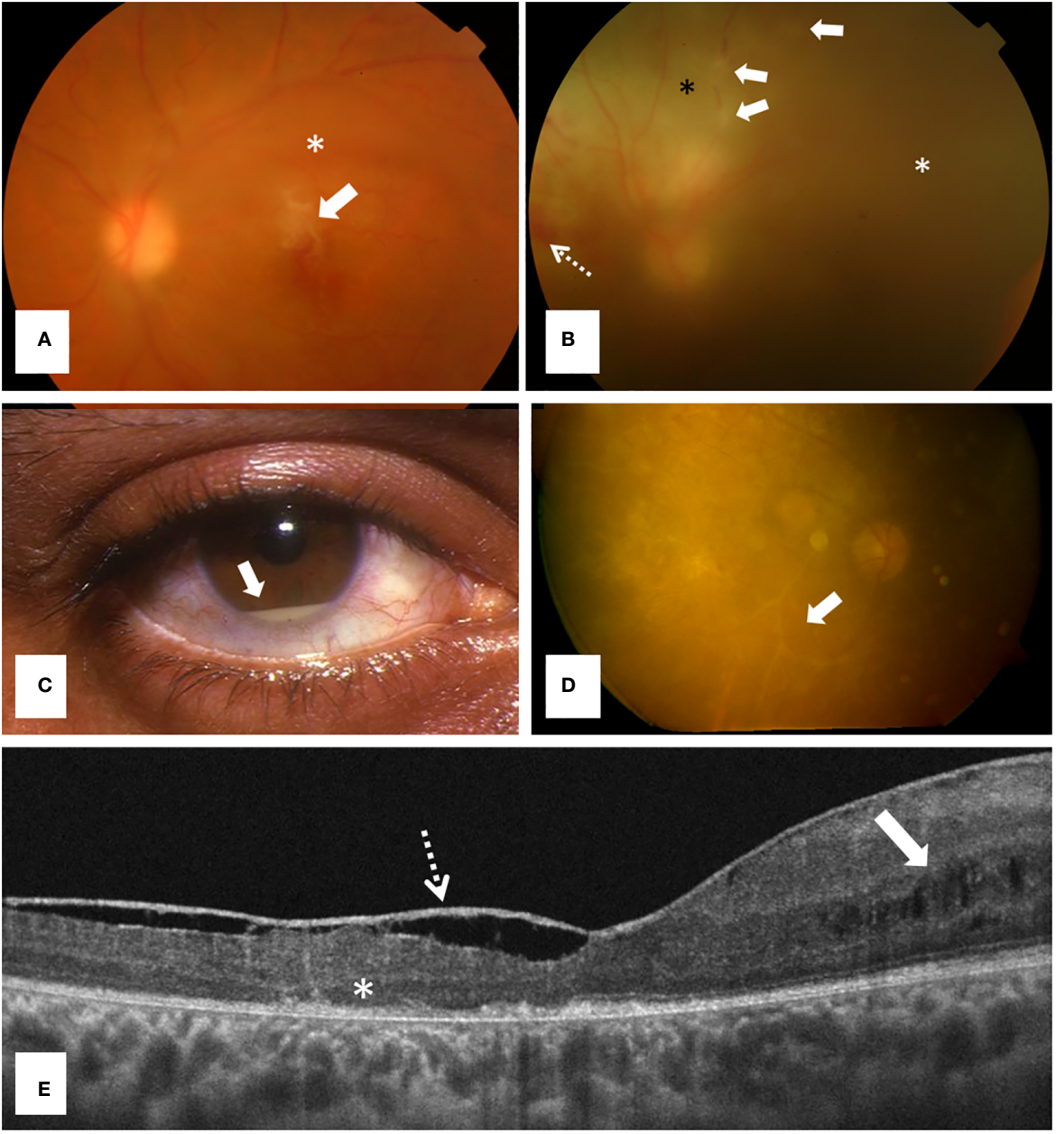
Ocular involvement in Behçet disease, a polygenic autoinflammatory disease. **(A)** Retinography showing a typical inflammatory infiltrate above the fovea (arrow) and mild vitritis (asterisk) in a 32 years old man consulting for acute loss of visual acuity; **(B)** Retinography revealing a retinal vasculitis with an ischemic area (black asterisk), phlebitis (arrows), retinal hemorrhages (dashed arrow) and severe vitritis (white asterisk) in a 21 years-old woman; **(C)** Anterior segment inflammation showing a hypopyon (arrow) (courtesy of Dr. Díaz-Valle); **(D)** Retinography detecting a severe retinal degeneration and ghost vessels (arrow) in a 32 years old male patient with long-term sequelae because he had only received glucocorticoids for managing panuveitis; and **(E)** Macular structural optical coherence tomography [in patient **(D)**] showing foveal atrophy, outer retinal abnormalities (asterisk), cystic changes in the inner nuclear layer (arrow) and a thickened posterior hyaloid (dashed arrow).

Regarding posterior segment involvement, the two main types of manifestations are retinal vasculitis and multifocal necrotizing retinitis (accompanied by varying degrees of vitritis) (**Figure 2A**). Retinal vasculitis, usually as phlebitis, exhibits the typical semiology of vascular cuffing, microhemorrhages and soft exudates (**Figure 2B**). Because major venous thrombotic events may occur due to the vascular inflammation and occlusion, extensive hemorrhages in the affected retinal territory may be concomitantly developed. Retinal artery occlusions are infrequent and show the typical whitish opacification of the involved retinal area (104). Necrotizing retinitis lesions are less frequently observed than retinal vasculitis and appear usually as yellowish or whitish infiltrates of variable size, depth and distribution (105–107).

The inflammatory involvement of the anterior segment (isolated or as part of panuveitis) is manifested as ciliary hyperemia, fine keratic precipitates, flare and anterior chamber cells. The presence of a hypopyon is considered a hallmark in Behçet’s disease uveitis (**Figure 2C**) (108). Hypopyon typically changes with gravity depending on patient’s head position. Fibrine and posterior synechiae develop in severe cases, and iris bombe normally results from chronic and recurrent inflammatory episodes (109).

Sclera may be also affected in Behçet’s disease, presenting as episcleritis or scleritis. While episcleritis may manifest with mild and even asymptomatic forms, and usually has an excellent visual prognosis, scleritis is a severe and potentially destructive disease, usually presenting with intense pain that may radiate to the periocular area (110). Other less frequent manifestations reported include keratitis, conjunctival ulcers, optic neuritis, oculomotor palsies, and orbital inflammatory disease (107).

Ocular complications are usually derived from the long-term inflammation in the affected eye segment and the prolonged treatment with glucocorticoids. Complications involving the anterior segment are mainly cataract and secondary glaucoma (109). In the posterior segment, complications include retinal and optic disc atrophy, retinal degeneration, ghost vessels (**Figure 2D**), cystoid macular edema, macular and foveal atrophy (**Figure 2E**), epiretinal membrane and full-thickness macular holes (105–107).

### Periodic fever, aphthous stomatitis, pharyngitis and cervical adenitis syndrome

PFAPA syndrome is the most frequent autoinflammatory disease in children. It is considered a polygenic or multifactorial condition because no associated genes or other etiologies have been found (111). Although etiopathogenesis of PFAPA syndrome is incompletely understood, IL-1 seems to play a biological role in the inflammatory response of this disease (112, 113). PFAPA syndrome may also occur in adult patients (18, 111, 114).

Clinical manifestations usually start in children under 5 years with acute febrile episodes of 3-6 days duration, with a mean frequency of 4 weeks. High fever is usually accompanied by the characteristic clinical triad that consists of oral aphthae, pharyngitis (or tonsillitis) and cervical adenitis (111). Rash, arthralgia, abdominal pain, nausea, diarrhea and headache are less frequent manifestations. Disease activity tends to improve over time and symptoms resolve after 4 to 8 years of the disease onset in most cases (111). Supportive pediatric classification criteria (115) and adult-onset diagnostic criteria for PFAPA syndrome (116) have been delineated. Several conditions, including undifferentiated and monogenic autoinflammatory diseases, such as HIDS and some forms of FMF, have to be included in the differential diagnosis.

Medium-to-high doses of glucocorticoids during 1 or 2 days at the beginning of the attacks are useful in abrogating the inflammatory reaction (9). Colchicine adds benefit in controlling disease flares in some patients. Tonsillectomy appears to be more effective in children than in adults (9). IL-1 blockers (anakinra and canakinumab) have provided good response in patients with PFAPA syndrome refractory to previous treatments (9, 117, 118).

#### Eye disease

Ocular inflammation in PFAPA syndrome rarely occurs as periorbital pain, conjunctivitis and intermediate uveitis (119, 120). Periorbital pain and conjunctivitis has been described in 2.4% and 12% of children, respectively, and in 16.7% and 13.3% of adult patients (120). Intermediate uveitis has been described in two patients of 7 and 8 years with typical changes at ophthalmologic examination, including vitreous cells, macular edema, and snow balls and snowbanking in the pars plana. Systemic glucocorticoids and methotrexate was used in both cases, and infliximab was finally administered with success in one of them. Both patients developed sight-threatening structural complications requiring surgical intervention, such as vitreous hemorrhage and tractional retinal detachment (119).

### Still’s disease

Still’s disease comprises two polygenic autoinflammatory diseases, now considered part of the age spectrum of the same disease: systemic juvenile idiopathic arthritis (sJIA) and adult-onset Still disease (AOSD). Both conditions share common pathogenic pathways, and IL-1β, IL-18, IL-6 and IL-17 are the some of the cytokines produced during Still’s disease flares (121).

Still’s disease, either in its pediatric or adult form, is similarly characterized by the presence of spiking fever, evanescent maculopapular erythematosus rash, arthralgia, abdominal pain, parenchymal lung disease, macrophage activation syndrome, and increased CRP, ESR and ferritin levels. By contrast, pediatric patients have been found to exhibit more frequently arthritis, and adult patients have a higher prevalence of extra-articular organ involvement, including sore throat, liver abnormalities, lymphadenopathy, splenomegaly, pericarditis and pleuritis, and leukocytosis (122). Several clinical phenotypes have been described in Still’s disease, either based on disease presentation over time (as monocyclic, polycyclic and chronic course) or according to the dominant clinical features (as systemic and arthritis subtypes) (121).

In real life, glucocorticoids and additional immunosuppressive drugs, mainly methotrexate, are usually used in mild articular forms (123). Biologic agents, such as IL-1, IL-6 and TNF blockers are used as first-line therapy more frequently in pediatric patients. Anti-IL-1 agents are similarly given to children and adults, and tocilizumab and TNF antagonists are more commonly chosen in adult and pediatric patients, respectively. Anakinra is the biologic agent more frequently administered, followed by etanercept, tocilizumab and canakinumab (122).

#### Eye disease

Ophthalmologic involvement in patients with pediatric and adult Still’s disease is really infrequent. Purtscher’s-like retinopathy, a rare condition presenting with retinal hemorrhages and prominent soft exudates due to ischemia probably secondary to complement-mediated vascular damage, and also associated with thrombotic microangiopathy, has been reported in several adult patients (124–128). Panuveitis has been described in a pediatric (129) and an adult patient (130). Other ocular lesions occasionally reported in adult patients with Still’s disease include scleritis (131), throcleitis (132), and orbital inflammation (133).

### Autoinflammatory bone diseases

Chronic recurrent multifocal osteomyelitis (CRMO) and synovitis, acne, pustulosis, hyperostosis and osteitis (SAPHO) syndrome are two rare autoinflammatory bone disorders characterized clinically by painful bone lesions and other common features (134). Due to their similarities, many authors consider CRMO to be a subtype of SAPHO syndrome. CRMO is the severe form of chronic non-bacterial osteomyelitis (CNO). The etiopathogenesis of these chronic diseases is not fully understood. However, genetic factors, infections, and immune dysregulation seem to play a role. An overproduction of IL-1β and dysregulation of IL-8, IL-17, IL-18, and TNF-α has been identified in SAPHO syndrome. The expression of the receptor activator nuclear factor κ-B ligand (RANKL) is increased in patients with active disease. In CRMO, inflammasome components, such as apoptosis-associated speck-like protein (ASC), NLRP3, and caspase-1, have been found elevated (134).

While CRMO tends to affect children, SAPHO syndrome generally occurs in adult patients. In both conditions, bones are affected mainly as polyostotic lesions, mainly the anterior chest wall, long bones and spine. Patients with SAPHO syndrome present with sterile joint inflammation (synovitis), cutaneous lesions (e.g. severe acne and palmoplantar pustulosis), hyperostosis and non-infectious osteitis. In CRMO/CNO, heterogeneous symptoms include fever, constitutional symptoms, severe bone pain and swelling of the affected area, usually worse at night (134).

Conventional treatments for SAPHO and CRMO include NSAIDs, glucocorticoids, additional immunosuppressive drugs, antibiotics, and bisphosphonates. In patients with CRMO, three treatment plans by consensus include sulfasalazine or methotrexate, anti-TNF agents (with optional methotrexate), and bisphosphonates. Among the latter, pamidronate has shown efficacy on osteoarticular manifestations in SAPHO syndrome, without impact on cutaneous lesions. TNF blockers, such as etanercept, adalimumab, and infliximab are generally used in patients with CRMO and SAPHO syndrome after methotrexate failure with good results. Anti-IL-1 agents have also shown efficacy in both diseases (134). JAK inhibitors and secukinumab, an IL-17RA inhibitor, provided good responses in patients with SAPHO syndrome (134–136). Denosumab, a human monoclonal antibody that binds to osteoblast-produced RANKL and inhibits osteoclastic-medicated bone resorption, has been also used with benefit in two patients with SAPHO syndrome (137, 138).

#### Eye disease

Ocular involvement as retinal vasculitis, optic neuropathy, central retinal artery occlusion and orbital inflammation has been reported in patients with CRMO (139–141). In patients with SAPHO syndrome, eye inflammatory lesions may occur as anterior scleritis, anterior uveitis, retinal vasculitis and Vogt-Koyanagi-Harada disease (142–146).

## Therapeutic approaches for ocular manifestations

Overall, the treatment of ocular manifestations in autoinflammatory diseases is the same as that followed for ocular involvement in other inflammatory or immune-mediated diseases, such as autoimmune diseases and systemic or localized vasculitis involving the eye. **Table 3** summarizes the main therapeutic approaches for ocular lesions in autoinflammatory diseases.

**Table 3:** Therapeutic approaches for ocular manifestations of autoinflammatory diseases.

Ocular manifestation	Treatment
Conjunctivitis/Epiescleritis	Topical nonsteroidal anti-inflammatory drugs or glucocorticoids
Scleritis	Systemic glucocorticoids
Uveitis	
*Anterior*	Topical glucocorticoids
*Intermediate/Posterior/Panuveitis*	Systemic/Periocular/Intravitreal glucocorticoids*
Keratitis/Dry eye	Lubricants, Topical glucocorticoids
Optic neuritis/Oculomotor palsies	Systemic glucocorticoids
Orbital inflammation	Systemic glucocorticoids
Specific eye complications	
*Choroidal neovascularization*	Intravitreal antiangiogenic agents
*Macular edema*	Systemic/Periocular/Intravitreal glucocorticoids*
*Retinal ischemia/neovascularization*	Laser photocoagulation

^*^In cases of unilateral involvement a local approach may be indicated, with periocular (subtenon injection) triamcinolone in non-severe lesions, and an implant of intravitreal dexamethasone in severe cases.

Acute ocular episodes are generally controlled with glucocorticoids, administered locally or systemically, depending on the location, laterality, and severity of the inflammatory lesions. In cases with maintained inflammation or repeated flares, refractory to initial glucocorticoids, long-term maintenance therapy with conventional immunosuppressive drugs and/or biological agents should be administered. The drug should be selected according to the knowledge or established guidelines for every systemic disease (e.g., the preferred medication for panuveitis in patients with Blau syndrome is based on TNF blockers, as they are the agents indicated for the systemic and ocular manifestations of Blau syndrome) (5).

Regarding structural complications potentially needing surgery, such as cataracts, the surgical procedure should be performed (if possible) in absence of ocular inflammation for at least 3 or 4 months, since it is well known that the invasive therapy on an inflamed eye may exacerbate the primary process and may be additionally associated with complications, such as macular edema or ocular hypotony (in severe cases). In order to avoid these postoperative complications, it is also recommended to administer perioperative periocular/intravitreal and/or systemic glucocorticoids. Finally, some ophthalmological complications may require specific management, such as intravitreal antiangiogenic drugs in cases of choroidal neovascularization, and laser retinal photocoagulation in patients with retinal ischemia and neovascularization (5).

## Conclusions and final remarks

Autoinflammatory diseases are rare conditions usually poorly recognized by non-specialized physicians and general ophthalmologists. The recognition of the systemic and ophthalmological manifestations of monogenic and polygenic autoinflammatory diseases is crucial to establish a correct diagnosis and to administer the most useful treatments as soon as possible.

In order to increase the awareness of general practitioners and ophthalmologists about the main monogenic and polygenic autoinflammatory diseases and their ocular manifestations, the most recent and updated data is herein reviewed.

## Author contributions

AF: Conceptualization, Writing – original draft, Writing – review & editing. EC: Validation, Writing – original draft. AV: Validation, Writing – review & editing. AJ: Validation, Writing – original draft. AR: Validation, Writing – original draft. LP: Validation, Writing – original draft. BS-Z: Methodology, Writing – original draft. VG-C: Validation, Writing – original draft. LC: Validation, Writing – original draft. CF: Validation, Writing – original draft. JH-R: Conceptualization, Validation, Writing – original draft, Writing – review & editing.
